# Minor Surgical Gynæcology

**Published:** 1881-01

**Authors:** 


### BIBLIOGRAPHICAL.

MINOR SURGICAL GYNÆCOLOGY. A manual of Uterine Diagnosis and the lesser technicalities of gynæcological practice, for the use of the advanced student and general practitioner. By Paul F. Munde, M.D., Professor of Gynæcology in Dartmouth Medical College, etc., etc.; with three hundred illustrations. Wm. Wood & Co., New York. 1880.

This is somewhat a novelty in specialties, but at the same time a very useful one apparently. Details of gynæcological practice that could not conveniently be discussed in general text-books are comprehensively presented in this work. Like works on minor surgery, in this we have descriptions of the instruments, dressings and the various manipulations and other detailed measures essential in gynæcology, and a knowledge of which is necessary in a successful gynæcological practice.

---

PAMPHLETS RECEIVED.—Scarlatina—A lecture in the Jefferson Medical College, by Wm. B. Atkinson, A.M., M.D., lecturer on Diseases of Children.

Rocky Mountain Health Resorts—An analytic study of high altitudes in relation to the arrest of chronic pulmonary disease, by Chas. Denison, A.M., M.D. Second edition. Riverside Press, Cambridge, 1881.

Some of the Errors in the Diagnosis of Eye Diseases into which general practitioners are apt to fall, by Sam'l Theobold, M.D., Surgeon to the Baltimore Charity Eye and Ear Infirmary. Reprint from *Maryland Medical and Surgical Journal*, for December 15, 1880.

